# Defining major trauma: a pre-hospital perspective using focus groups

**DOI:** 10.29045/14784726.2019.12.4.3.16

**Published:** 2019-12-01

**Authors:** Lee Thompson, Michael Hill, Peter McMeekin, Gary Shaw

**Affiliations:** North East Ambulance Service NHS Foundation Trust; Northumbria University; Northern Trauma Network: ORCID iD: https://orcid.org/0000-0002-0820-1662; Northumbria University; Northumbria University; North East Ambulance Service NHS Foundation Trust

**Keywords:** elderly, focus group, injury severity score, mechanism of injury, older adult, trauma

## Abstract

**Background:**

Pre-hospital trauma is complex and challenging, with limited clinical exposure for clinicians. In addition, there is no standardised definition for major trauma, and retrospective scores commonly quantify injury severity, such as the injury severity score. This qualitative study aimed to explore the pre-hospital perspectives of major trauma and how pre-hospital trauma care providers define major trauma.

**Method:**

Three focus groups of 40–60 minutes’ duration were conducted with paramedics, ambulance technicians, police, firefighters and emergency dispatchers. Digital recordings were transcribed verbatim, coded and reviewed to identify emerging themes. Constant comparison was undertaken throughout and codes were identified for qualitative thematic analysis.

**Results:**

Three overarching themes emerged: clinician factors, patient factors and situational factors. Clinician factors highlighted issues of experience and exposure (or lack of) to major trauma and its relationship to clinical concern, communication issues and the complex nature of pre-hospital trauma. Patient factors identified deranged physiology, actual injuries, life changing trauma, potential need for surgical intervention and rehabilitation as defining major trauma. These variables are often complicated by the extremities of age as well as previous medical history and medications. The situational factors identified that every traumatic encounter is unique, requiring bespoke management where high and low energy mechanisms of injury should be considered.

**Conclusion:**

Based on the analysis of the focus groups, a working pre-hospital definition is: Any injury (or injuries) that have the potential to be life-threatening or life-changing, including those sustained from low energy mechanisms in people rendered vulnerable by extremes of age, comorbidities or frailty, resulting in significant physiological compromise (haemodynamic instability, reduced consciousness, respiratory compromise) and/or significant anatomical abnormality that may require immediate intervention.

## Introduction

### Problem formulation

The pre-hospital environment is complex and challenging and, with the exception of specialised teams, exposure to major trauma is very rare. Although major trauma is the leading cause of death in the United Kingdom for adults under 40 years (Moran et al., 2018), there is a noticeable shift in what was once thought of as a younger adults’ disease. Kehoe, Smith, Edwards, Yates and Lecky (2015) highlight that major trauma patients are now more likely to be elderly and sustain significant injuries from relatively minor mechanisms of injury (MOI). From our own experiences within the Northern Trauma Network (NTN), MOI and injury severity do not correlate well – a finding corroborated elsewhere in the literature (Magnone, Ghirardi, Ceresoli, & Ansaloni, 2019; Potter, Kehoe, & Smith, 2013; Stuke et al., 2013).

**Figure 1:**
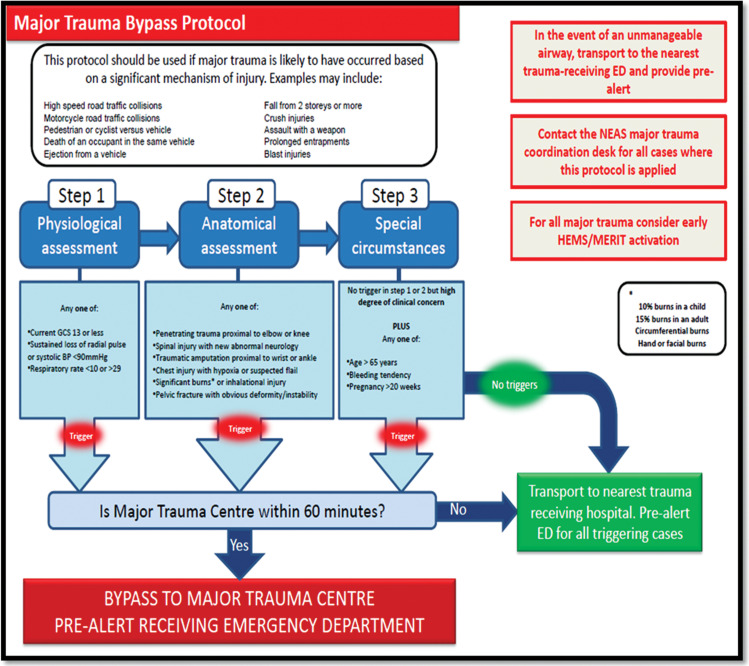
Figure 1. Example of a major trauma bypass protocol.

It is important to identify patients needing transport to a major trauma centre (MTC) for definitive care, as this has been associated with improved outcomes (Moran et al., 2018). UK ambulance services use a number of major trauma triage tools (Figure 1). However, a lack of consensus exists in relation to what constitutes a useful or standard definition of major trauma (Alberdi, Garcia, Atutxa, Zabarte, & Trauma and Neurointensive Care Work Group of the SEMICYUC, 2014). One approach involves retrospective scoring such as the injury severity score (Thompson, Hill, & Shaw, 2019). Unfortunately, injury scoring systems are often calculated after all imaging and interventions are completed, making them unsuitable for the pre-hospital phase of care.

### Aims

This study aimed to explore the perspectives and definitions of major trauma among a sample of pre-hospital trauma care providers, including NHS and non-NHS emergency services and first responders. The research question was: In the absence of retrospective scoring systems, what considerations do pre-hospital care providers apply to defining major trauma?

## Methods

### Qualitative approach and research paradigm

We undertook three focus groups with a total sample of 45 NTN pre-hospital trauma care providers. Focus group research entails organised discussion(s) with a selected group of individuals to gain information about their views and experiences of a topic in which the key data output is interaction between participants (Morgan, 1997). The primary reason for using a focus group approach in this instance was as an attempt to capture collective thoughts, feelings and experiences in relation to working definitions of major trauma in pre-hospital care. Race, Hotch and Packer (1994) asserted that focus groups are a particularly useful methodology for obtaining several perspectives about a topic and investigating collective understandings of a concept.

Each focus group was undertaken in a different location to capture any potential geographical idiosyncrasies, and was conducted using semi-structured questions lasting approximately 40–60 minutes. The conversations were digitally recorded and transcribed verbatim. Anonymity was maintained during transcription by utilising codes allocated to each participant.

### Researcher characteristics and reflexivity

The researchers conducting the focus groups (LT and GS) are specialist paramedics with 25 years’ experience working within the North East Ambulance Service (NEAS) and are currently part of a dedicated trauma team which is unique to NEAS. Both are familiar with the research setting but not necessarily familiar with all research participants. Continuous reflection was undertaken, along with peer debriefing with the research team and participant checking to enhance trustworthiness of the data and analysis.

### Context

The NTN is made up of eight trauma units and two MTCs covering North Cumbria and North East England, from the Scottish Borders to Yorkshire. NEAS is the main ambulance service provider operating within the NTN which also has two Helicopter Emergency Medical Service (HEMS) bases and two other ambulance service providers: Yorkshire Ambulance Service and North West Ambulance Service. NEAS covers 8365 km^2^ and receives over 1.5 million urgent and emergency calls per year. There is a mixed geography of densely populated cities and remote rural locations where community volunteers, police and fire and rescue also co-respond to emergency calls.

### Sampling strategy

A purposive sample (Etikan, Musa, & Alkassim, 2016) of pre-hospital trauma care providers was recruited via email and social media. Those expressing an interest in participating were then screened by virtue of their knowledge and experience. No exclusions were placed on age, experience or area of practice.

While being aware of tensions around what constitutes ‘best practice’ in the conduct of focus groups with respect to sample size and group homogeneity (Freeman, 2007), we adopted a pragmatic approach, running focus groups of 15 participants and taking a flexible attitude to group composition. Larger groups can be difficult to moderate; however, in this study, all of the groups worked well and resulted in enlightening discussions and data.

There is also a clear lack of guidance or recommendation as to the length of focus group interviews. Within the context of our study it was believed that three focus groups lasting approximately 60 minutes would provide data for analysis, but if saturation was not achieved further iterations would be undertaken.

### Data collection method

A semi-structured interview format was followed, chosen on the basis that it is well suited for the exploration of perceptions and opinions of participants regarding complex matters and potential to probe for further information or clarification (Bryman, 2016) (Supplementary 1). The varied professional, educational and personal histories of participants precluded the use of a standardised interview schedule and the semi-structured format accommodated these differences.

### Data analysis

Analysis began after the first focus group which influenced and ran concurrently with each iteration of the focus groups.

The transcripts were managed and explored with NVivo qualitative data analysis software, QSR International Pty Ltd., Version 11, 2015. Coding was undertaken and reviewed to identify emerging theoretical and conceptual themes (Saldaña, 2013). As data were collected concurrently, constant comparison was undertaken throughout and new codes identified for qualitative thematic analysis. The data analysis framework approach recommended by Pope, Ziebland and Mays (2000) was used throughout the study.

## Results

Between February and March 2018, three focus groups were undertaken and a total of 45 participants attended (Table 1). All participants work within the NTN region and were exposed to trauma within the context of their individual roles and were familiar with the major trauma bypass (triage) protocol. Participants were predominantly NEAS paramedics, which included specialist Hazardous Area Response Team paramedics. Also included were HEMS (and former HEMS) paramedics, emergency dispatch officers, call takers, voluntary sector ambulance technicians, police authorised firearms officer tactical team medics and fire and rescue firefighters. Participants varied on the basis of age, role and experience. Route of entry into the paramedic profession was not ascertained for those paramedics who attended. Experience (for clinicians) ranged from less than one year’s front line experience (post qualification/registration) to over 25 years’ experience (mean of 12.5 years calculated for a single focus group only). There were limited numbers of sub-groups of participants; therefore, to minimise identifying individuals, sub-groups were labelled as: paramedic, non-UK trained paramedic, non-registered clinician and non-clinician.

**Table 1.** Focus group participants.

Role	n (%)
Paramedic (NHS)	33 (73)
Ambulance technician/care assistant (NHS)	4 (9)
Ambulance technician (non-NHS)	2 (4)
Police	2 (4)
Firefighter	2 (4)
Emergency dispatcher/call taker	2 (4)
Total participants	45 (100)

There were three overarching themes when participants were determining the definition of major trauma: clinician factors, patient factors and situation factors.

### Clinician factors

There were five overlapping sub-themes that emerged from the clinician factors identified during the focus groups (Table 2).

**Table 2.** Clinician factors in defining major trauma.

Sub-theme	Factor
Experience	Exposure to (or lack of) Identifying injuries and ongoing care needs Specific patient group needs Intuition/instinct Includes all the other themes below
Clinical concern	Linked to experience and potential injuries
Difficulties	Communication issues Environmental factors Adrenaline rush (effecting decision making) Distracting factors Limited information
Index of suspicion	Based on MOI and potential for injury
Potential for injury	Suspicion based on experience and MOI and assessment

MOI = mechanisms of injury.

The overarching theme is based on a clinician’s experience, exposure to trauma and how they instinctively/intuitively identify major trauma.

**Paramedic A:** … you’ve just got to use your clinical judgement at times … you have just relied upon your experience and your knowledge and understanding … your gut instinct is always the best instinct …

**Paramedic B:** It’s a big worry … but I [think] nine times out of ten your gut feeling is normally the right one.

It was acknowledged that a high degree of clinical concern, based on experience and exposure, influences clinical decision making when identifying potential major trauma patients. Multiple factors are considered and, therefore, each patient is unique and should be managed as such with bespoke care.

**Paramedic C:** Every life matters … individual care, bespoke care … every person is different.

A consensus highlighted that there is a balance between knowledge and experience when identifying major trauma patients. It was suggested that a high degree of clinical concern is not often considered when discussing patients with emergency departments (EDs) who do not meet the strict criteria for direct access to the MTC.

A number of factors influence a clinician’s index of suspicion and it is based on the unique circumstances (and available/limited resources) presented at that moment in time, with multiple difficulties and variables which form their perception of the situation. Examples included communication issues such as: the dementia patient, the child that has not yet acquired language skills, intoxicated patients, non-verbal patients or those whose primary language is different from the clinician’s.

Being able to communicate with colleagues via the emergency operations centre or directly with the MTC assisted in identifying those individuals who should be managed as major trauma, although this was not always a good solution:

**Paramedic D:** … the clinician [at other end of phone] may still be really inexperienced … and getting through to them … is quite difficult.

### Patient factors

Several themes discussed within all the focus groups centred on patient factors when it came to defining major trauma (Table 3).

**Table 3.** Patient factors in defining major trauma.

Sub-theme	Factor
Physiology	Altered physiology
Outcome measures	Injuries Life changing Need for surgical intervention Rehabilitation
Pre-trauma factors	Age Previous medical history Medications Co-morbidities

It was widely accepted that patients who experience any form of trauma and have deranged physiology (reduced consciousness, falling blood pressure or altered respiratory rate/pattern) should be managed as major trauma. However, it was also noted to be helpful to know the patient’s normal physiological parameters (prior to trauma) so comparisons could be made, and consequential management determined. In addition, awareness of the patient’s previous medical history and medications was also implicated in influencing ongoing management (e.g. prescribed medications that increase the likelihood of haemorrhage or make the patient susceptible to injury).

**Paramedic E:** … again, individual care … specific to that person. So, the 12-year-old who’s had the same mechanism got the same presentation but the little nanna who’s on ten drugs, osteoporotic, curvature of the spine and stuff. You’re going to kind of manage them … a bit … [differently].

One of the main themes discussed was specific age groups. It was obvious within the age group discussions that trauma involving children is very emotive. Specific paediatric trauma triage tools were also discussed; however, most clinicians stated that they were inclined to rapidly transport paediatric trauma to the MTC without referring to a trauma triage tool. This again relates to a lack of exposure to paediatric trauma (and over triage).

**Paramedic F:** … they could sit there looking a bit alright but have come out of this awful mechanism, you wouldn’t want to just say they are probably fine, they look alright, they are probably fine [but] you would want them to be seen.

**Non-UK trained paramedic:** The problem is that because the ambulance service is exactly the same in [country] as they over triage the kids.

**Paramedic G:** [in relation to rare exposure to paediatrics] … you need that expert advice to start with [via MTC direct line]. So you are making those correct decisions.

Discussions relating to older adults and frail patients identified that a significant volume of trauma cases involved older adults who have relatively minor MOIs with no obvious significant injury at the time of incident, but are later diagnosed with significant injuries. It was acknowledged that this is a challenging group as there is a real risk of overwhelming EDs with older adults who have simple falls with potential injuries.

**Paramedic H:** Age, it doesn’t matter … It’s all about the injury or potential injury.

**Paramedic I:** I think your index of suspicion is going to be higher [in the elderly]. They have a potential for more to be wrong and not show any symptoms compared to younger ones.

**Paramedic J:** … stabbings for example … you ask for an enhanced care team … helicopters … come out because, it’s a sexy trauma. It’s [name] who has fallen in the back garden … they are not going to come out to her.

A surprisingly minor focus of discussion regarding patient factors were the actual patient outcomes. These seemed to be, in the main, identifiable injuries – those injuries that are life or limb threatening or require rehabilitation. Observing these discussions, it was easy to conclude that the clinicians were happy to define patients as major trauma where obvious significant injuries were identified. This included injuries that would require interventions (surgical or otherwise) within their ongoing journey of care.

What made defining major trauma difficult was the subtle or occult injuries that may later develop. This was further complicated when talking about the older or frail trauma patient. There was also a real concern for missing potential injury that is not apparent in the initial assessment.

### Situation factors

Several themes discussed within all focus groups centred on situational factors (Table 4).

**Table 4.** Situation factors in defining major trauma.

Sub-theme	Factors
Bespoke	Every patient, environment, situation is unique and requires a bespoke management plan
MOI	Low energy High energy Influence of alcohol
Triage	Tools have a role to play Triage tools make generalisations and potentially miss many patients

MOI = mechanisms of injury.

There were some enlightened discussions throughout the focus groups which commented upon individual factors that need to be placed into context and that when combined are often greater than the sum of their individual parts.

**Paramedic K:** You need a holistic view to see everything … It’s individual to that patient [and] how they present to you at that moment.

**Paramedic L:** Every person is different … you can have the same injury on two different people and that body will react in different ways.

The consensus of the discussions was to provide individual care for a specific patient at a specific time, combining multiple factors to define that patient as major trauma.

MOI was an interesting area of discussion where all groups stated that high energy mechanisms heightened their suspicion of injury but that traumas from minor mechanisms were equally important as they may cause significant injury.

**Paramedic M:** … mechanism is something you need to consider. And you need to take [it] into account …

**Paramedic N:** … the 16-year-old fallen over is probably going to bounce and get up, but an 82-year-old might have a serious injury due to underlying medical conditions …

When discussing triage, the use of triage tools was prominent in highlighting that many patients who the clinicians believed were major trauma did not fit within the parameters of the major trauma triage tool.

**Paramedic O:** … the trauma triage tool has a role to play but it’s not necessarily accurate … [in identifying all major trauma] …

**Paramedic P:** [the major trauma triage tool is] not the be all and end all; it is about the suspicion of injury …

**Non-clinician:** from a personal perspective I had a few injuries a couple of years ago. And I don’t see on [the major trauma triage tool] that I would be [major trauma], and would have thought I was majorly traumatically injured.

**Paramedic Q:** [pointing to triage tool] … it’s a good starting point … You don’t necessarily have to agree with it. But it is a good prompt … [and] a good reference when you’re in a high stress situation.

## Discussion

### Key results

It was obvious that experience plays a significant role in identifying and managing major trauma. Weiss et al. (2018), when discussing out-of-hospital cardiac arrests, highlighted that experience and exposure have a direct influence on outcomes in rare and stressful incidents. This suggests that the use of specialist teams who are regularly exposed to, and train for, major trauma have a role within ambulance services to improve outcomes for this patient group.

The lack of exposure to trauma by many clinicians may influence clinical decision making, which in turn is complicated by patient factors such as age, MOI, previous medical history and altered physiology. It is apparent that our understanding of major trauma in England is changing and there is a need to improve our identification and management of the older adult who experiences major trauma from relatively minor MOIs such as a fall from standing height (Kehoe et al., 2015).

Children experiencing major trauma is a rare event, and of those who are severely injured (n = 1511 between January 2013 and December 2014 in England and Wales), only 56% are transported by ambulance services (Trauma Audit & Research Network, 2015). It is understandable, therefore, that ambulance clinicians are anxious about managing these highly emotive cases. Although paediatric trauma is emotive and often over triaged, there is clear need for guidance within high stress environments to minimise human error by obtaining skilled advice remotely and/or using age specific trauma triage tools.

Triage tools (checklists) have an acknowledged role to play and may provide reassurance for clinicians when dealing with rare events that are potentially highly stressful (Clay-Williams & Colligan, 2015). However, these tools are very poor in identifying older adults who have significant injuries from low MOI (Potter et al., 2013). Trauma within the United Kingdom is changing, as the older adult is now the emerging focus of major trauma (Kehoe et al., 2015). As such, bespoke older adult triage tools need to be developed to identify those older adults who need early intervention.

It is also acknowledged that, in isolation, MOI is a very poor indicator of outcome and should only be used in identifying major trauma when other factors, such as deranged physiology, are present (Boyle, 2007). If MOI is used in isolation it is likely to over triage major trauma (Lossius et al., 2001; Magnone et al., 2019). We have a culture of using MOI as an indicator of trauma, and this is perhaps no longer appropriate and should be addressed within academia and clinical practice. Linking in with age specific and bespoke care, Magnone et al. (2019) recommend that when utilising MOI, the older trauma patient should have an age specific triage protocol to assist in identifying those who require specialist interventions.

### Limitations

The participants all worked within a single trauma network and therefore their views may not be transferable but should be generalisable to any pre-hospital provider within a trauma network. Within group discussions there is a risk that single participants can dominate the group and therefore bias the views of others within the group. It is believed that having more than one focus group that was well facilitated will have minimised any individual dominating and biasing the data collected.

Focus group discussions present the participants’ view of reality and there may be differential understandings and perspectives between researcher and participant. Within the context of this research, the lead researcher (LT) is an experienced paramedic, which should have minimised misinterpretation of the data. Initial transcription and original coding and interpretation were cross checked by another experienced paramedic who was present during all focus groups. However, as an experienced paramedic, the researcher who facilitated the focus groups may have unintentionally biased the content and direction of the discussions. To minimise the risk of researcher bias, semi-structured questions were used to focus the content of each group discussion.

## Conclusions

Major trauma is unique to every provider, patient and situation that requires a bespoke management strategy. While MOI can raise the index of suspicion that major trauma has occurred, minor mechanisms, such as a fall from standing height, should not be discounted when identifying major trauma. There are challenges with accurately triaging patients at either end of the age spectrum, making the development of age-specific triage tools a focus for future research.

In the absence of retrospective scores, and based on using the data from the focus groups, we propose the following pre-hospital definition of major trauma: Any injury (or injuries) that have the potential to be life-threatening or life-changing, including those sustained from low energy mechanisms in people rendered vulnerable by extremes of age, comorbidities or frailty, resulting in significant physiological compromise (haemodynamic instability, reduced consciousness, respiratory compromise) and/or significant anatomical abnormality that may require immediate intervention.

## Acknowledgements

The researchers acknowledge the contributions of Fiona Lecky for her mentorship throughout this project, and Wilma Harvey-Reid for her valuable time in proofreading and manuscript layout.

## Author contributions

LT: Lead author, focus group facilitator, data collection, analysis and interpretation of data/literature.

MH: Contributing author, mentor and analysis and interpretation of data/literature.

PM: Contributing author, mentor and analysis and interpretation of data/literature.

GS: Contributing author, focus group facilitator, peer discussion, analysis and proofreading.

All authors have read and approved the final manuscript.

## Conflict of interest

None declared.

## Ethics

The protocol for the study was reviewed and approved by Northumbria University Ethics Committee (ref. 5714). Each participant received an information sheet prior to the focus group and consented on the day. No financial/gift incentives were offered, but training and education in advanced trauma care was provided along with refreshments during the session.

## Funding

This study was funded by a small research grant from the College of Paramedics.
